# Conserved Roles of the Prion Protein Domains on Subcellular Localization and Cell-Cell Adhesion

**DOI:** 10.1371/journal.pone.0070327

**Published:** 2013-07-31

**Authors:** Gonzalo P. Solis, Yvonne Radon, Emily Sempou, Katharina Jechow, Claudia A. O. Stuermer, Edward Málaga-Trillo

**Affiliations:** Department of Biology, University of Konstanz, Konstanz, Germany; INRA, France

## Abstract

Analyses of cultured cells and transgenic mice expressing prion protein (PrP) deletion mutants have revealed that some properties of PrP -such as its ability to misfold, aggregate and trigger neurotoxicity- are controlled by discrete molecular determinants within its protein domains. Although the contributions of these determinants to PrP biosynthesis and turnover are relatively well characterized, it is still unclear how they modulate cellular functions of PrP. To address this question, we used two defined activities of PrP as functional readouts: 1) the recruitment of PrP to cell-cell contacts in *Drosophila* S2 and human MCF-7 epithelial cells, and 2) the induction of PrP embryonic loss- and gain-of-function phenotypes in zebrafish. Our results show that homologous mutations in mouse and zebrafish PrPs similarly affect their subcellular localization patterns as well as their *in vitro* and *in vivo* activities. Among PrP’s essential features, the N-terminal leader peptide was sufficient to drive targeting of our constructs to cell contact sites, whereas lack of GPI-anchoring and N-glycosylation rendered them inactive by blocking their cell surface expression. Importantly, our data suggest that the ability of PrP to homophilically *trans*-interact and elicit intracellular signaling is primarily encoded in its globular domain, and modulated by its repetitive domain. Thus, while the latter induces the local accumulation of PrPs at discrete punctae along cell contacts, the former counteracts this effect by promoting the continuous distribution of PrP. In early zebrafish embryos, deletion of either domain significantly impaired PrP’s ability to modulate E-cadherin cell adhesion. Altogether, these experiments relate structural features of PrP to its subcellular distribution and *in vivo* activity. Furthermore, they show that despite their large evolutionary history, the roles of PrP domains and posttranslational modifications are conserved between mouse and zebrafish.

## Introduction

The prion protein is a cell surface glycoprotein expressed in many cell types, particularly in the nervous system. Its propensity to misfold and aggregate is central to the pathogenesis of transmissible spongiform encephalopathies (TSEs). Interestingly, the physiological role of PrP and its connection to prion neurotoxicity remain open questions. Although PrP knockout mice were initially found to be normal [1], [2], more recent analyses have uncovered PrP phenotypes related to the maintenance of peripheral myelin, olfactory physiology, neural precursor proliferation, adult neurogenesis, neurite elongation and muscle regeneration [3]–[6]. Further studies suggest that the mechanistic basis of these functions is the ability of PrP to modulate intracellular signaling [7]–[12].

**Figure 1:**
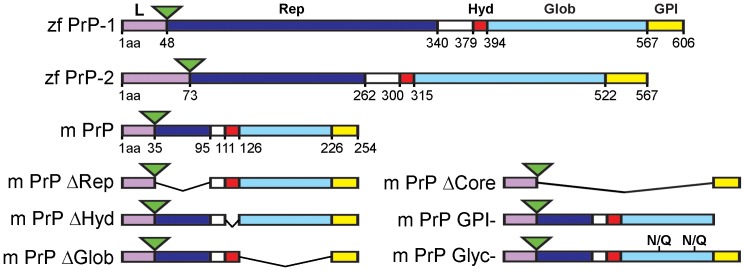
EGFP-tagged PrP constructs used in this study. The structural domains of zebrafish (zf) PrP-1, PrP-2 and mouse (m) PrP are represented as follows: leader peptide containing the polybasic motif (L) in violet, repetitive domain (Rep) in blue, hydrophobic region (Hyd) in red, globular domain (Glob) in light blue and GPI-anchored signal (GPI) in yellow. Amino acid (aa) positions of mouse and fish PrP domains are indicated. The EGFP fluorescence tags are depicted as green triangles. Deletion constructs lacking Rep (ΔRep), Hyd (ΔHyd), Glob (ΔGlob), Rep+Hyd+Glob (ΔCore), GPI (GPI^−^) and N-glycosylation sites (Glyc^−^) are shown for mouse PrP only. PrP domains were defined by evolutionary criteria [13].

Fish and mammalian PrPs share a common protein domain organization (Fig. 1) [13], [14]: A flexible N-terminal half (repetitive domain) and a well-structured C-terminal half (globular domain) connected by a short and highly conserved stretch (hydrophobic region). During biosynthesis, the immature polypeptide undergoes the cleavage of an N-terminal signal peptide and becomes tethered to the plasma membrane via the addition of a C-terminal glycosyl-phosphatidylinositol (GPI) anchor. Within the globular domain, formation of one disulfide bond and attachment of two N-linked oligosaccharide chains take place.

The relative contributions of each of these protein features to the cellular biology of PrP have been extensively studied in cultured cells and transgenic mice. For instance, at the N-terminus, the leader peptide is required for targeting to the ER [15], [16], whereas the adjacent polybasic motif has been reported to interact with glycosaminoglycans (GAGs) and influence the clathrin-dependent endocytosis of PrP [17]–[20]. More recently, mouse residues 23–31 have emerged as a key region controlling the neuroprotective activity of PrP and the neurotoxicity of a PrP mutant lacking the central region [21]–[23]. The repetitive domain appears to mediate copper-induced endocytosis of PrP and its association to lipid rafts [19], [24], [25]. The central hydrophobic region modulates a neurotoxic activity, recently connected to the generation of ionic currents [26]–[28]. The globular domain, on the other hand, has not been assigned any molecular roles other than serving as a template for prion replication. Interestingly, mutation of N-glycosylation sites impairs PrP transport to the plasma membrane and confers it with biochemical prion-like properties [29], whereas removal of the GPI-anchor turns PrP into a largely unglycosylated, secreted molecule [30]. Finally, both GPI-anchoring and N-glycosylation regulate the polarized sorting of PrP in epithelial cells [31], [32].

Recently, fish have emerged as alternative models to study the roles of PrP in health and disease [33]. Through combined experimental approaches in zebrafish embryos and cells in culture, we identified roles of PrP-mediated signaling in the regulation of embryonic cell adhesion [34]–[36]. In particular, we used *Drosophila* S2 and mouse N2a cells to show that vertebrate PrPs accumulate at cell-cell contacts, where they directly promote cell adhesion and protein phosphorylation via Src family kinases (SFKs). In early zebrafish embryos, similar PrP-dependent signals further regulate the stability of adherens junctions by modulating the transport of E-cadherin to/from the plasma membrane. More recently, we found that in A431 -a human epithelial carcinoma cell line- downregulation of PrP disturbs the formation of adherens junctions [37]. Here we sought to analyze how the protein domains and posttranslational modifications of PrP contribute to its localization and role at cell contacts in *Drosophila* and mammalian cells, as well as in the zebrafish embryo.

## Materials and Methods

### Molecular Cloning

The wild type (WT) mouse and zebrafish EGFP-PrP and corresponding EGFP-GPI control constructs have been previously described [34]. All deletion mutants used in this study (Fig. 1) were engineered by overlap extension PCR followed by insertion into the BglII/EcoRI sites of the corresponding EGFP-WT PrP vectors. The amino acid deletions within mouse and zebrafish PrP sequences are located as follows: repetitive (Δ36–95, Δ49–340 and Δ74–262 for mouse PrP and zebrafish PrP-1 and -2, respectively), hydrophobic (Δ112–126, Δ380–394 and Δ301–315), globular (Δ127–226, Δ395–567 and Δ316–522), core (Δ36–226, Δ49–567 and Δ74–522), and GPI (Δ227–254, Δ568–606 and Δ523–567). These exact positions correspond to evolutionarily conserved regions of PrP based on fish-to-mammal sequence comparisons [13]. The mouse PrP N-glycosylation mutants were engineered by introducing point mutations (asparagine to glutamine) at residues 180 and 196. The zebrafish PrP N-glycosylation mutants have been reported previously [34]. For transfection into *Drosophila* S2 cells, EGFP-PrP constructs were subcloned into the XbaI/ApaI sites of the pAc5.1/V5-HisA vector (Invitrogen). For expression in zebrafish embryos, EGFP-PrP constructs were subcloned into the EcoRI site of pCS2+ and transcribed *in vitro* (see below).

### Cell Culture and Transfection

MCF-7 cells (ATCC) were maintained in 10% FCS MEM (Invitrogen), supplemented with L-glutamine and penicillin-streptomycin at 37°C and 5% CO_2_. Cells were grown on poly-lysine (pLys) coated coverslips for 24 h prior to transient transfection using Lipofectamine 2000 (Invitrogen). S2 cells (Invitrogen) were maintained in 10% FCS Schneider’s Medium (AMIMED), supplemented with L-glutamine and penicillin-streptomycin at 24°C. Cells were grown for 24 h prior to transient transfection using Effectene (QIAGEN). Analyses were performed 24 h after transfection.

### Cell Contact Formation Assays in Drosophila S2 Cells

After transfection with mouse or zebrafish EGFP-PrP vectors for 24 h, S2 cells were incubated in PBS supplemented with 0.05% trypsin for 5 min at RT. After washing, cells were resuspended in 10% FCS Schneider’s medium and incubated for 2 h before mounting. Alternatively, trypsinized cells were incubated in standard medium for 1 h previous to treatment (0.5 or 1 h) with 10 µM Cytochalasin D, 5 µM Nocodazol, 50 µM PP2 (all Calbiochem) or DMSO as control. Cells were then mounted for quantification as described previously [34]. At least ten low-magnification fields of equal cell density were randomly taken from each experiment using Plan-NEOFLUAR 40×objectives and an AxioCam HRm on an Axioplan 2 microscope (all Zeiss). Cell contacts exhibiting accumulation of the corresponding EGFP-PrP construct were quantified and given as the percentage of total cell contacts made by transfected cells (average ± SEM; *n* = 3, ∼200 cell contacts per experiment; one-way ANOVA test).

### Immunostaining of MCF-7 Cells

MCF-7 cells were grown on pLys-coated coverslips, fixed for 15 min in 4% PFA 24 h after transfection, permeabilized with 0.1% Triton X-100 in PBS and probed for 1 h at RT with primary monoclonal antibody against E-cadherin (BD Bioscience, 1∶1000 dilution) followed by incubation in 1∶1000 diluted Cy3-conjugated donkey anti-mouse secondary antibody (Jackson ImmunoResearch) and DAPI also for 1 h at RT. Cells were recorded using a Plan-Apochromat 63×/1.4 objective in a confocal laser-scanning microscope (LSM510 META, Zeiss).

### Zebrafish

Wild type adult zebrafish were maintained and crossed at the University of Konstanz’s animal facility using previously established methods [38]. Freshly fertilized embryos were incubated at 28.5°C in E3 medium (5 mM NaCl, 0.17 mM KCl, 0.33 mM CaCl_2_, 0.33 mM MgSO_4_) and staged according to [39].

### mRNA Synthesis, Overexpression and Rescue Experiments

In order to generate capped mRNAs for microinjection, 1 µg of each construct’s DNA was linearized and used as a template for *in vitro* RNA synthesis reactions (mMessage mMachine SP6 kit, Ambion). To express PrP constructs in zebrafish embryos, the corresponding mRNAs were titrated and microinjected as previously described [34]. Gastrulation phenotypes were scored for an average of 30 embryos per sample in three independent experiments. The percentage of embryos showing abnormal phenotypes were statistically confirmed using one-way ANOVA tests. Live images were taken on a LUMAR.V12 stereomicroscope (Zeiss) and further processed using Adobe Photoshop CS5.

### Zebrafish Immunostainings

Zebrafish embryos were fixed and stained as previously described [34] using the following antibodies: mouse monoclonal anti E-cadherin (BD Biosciences, 1∶1000 dilution), rabbit polyclonal anti pY416-Src (Cell Signalling, 1∶250 dilution) and mouse monoclonal anti β-catenin (BD Biosciences, 1∶250 dilution). Cy3-conjugated donkey anti-rabbit (Jackson Immunoresearch, 1∶1000) and Cy5-conjugated goat anti-mouse (Invitrogen, 1∶1000 dilution) were used as secondary antibodies. Embryo flat mounts in were prepared in 80% glycerol/PBS and visualized on an LSM 510 confocal microscope (Zeiss). Images were further processed using Adobe Photoshop CS5.

## Results

### PrP-dependent Cell Contact Formation in Non-adhesive Drosophila S2 Cells

**Figure 2:**
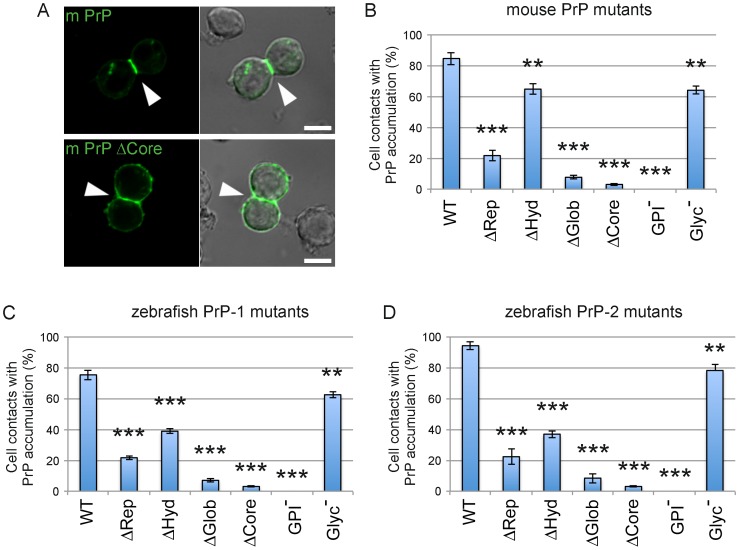
Accumulation of mouse and zebrafish PrPs at newly formed cell contacts in *Drosophila* S2 cells. A) Expression of the mouse PrP EGFP fusion construct (m PrP) induces cell contact formation and the subsequent accumulation of PrP at contact sites. This is not observed at fortuitous contacts formed by cells expressing a control construct lacking the major PrP domains (m PrP ΔCore). Cell-cell contacts are indicated by white arrowheads. Scale bars = 5 µm. B-D) Quantification of the number of S2 cell contacts showing accumulation of wild type (WT) and mutant constructs for mouse PrP (B), zebrafish PrP-1 (C) and zebrafish PrP-2 (D). Construct names are inserted in the graphs. Double and triple asterisks [** and ***] indicate statistical significance at *p*<0.01 and <0.001, respectively; one-way ANOVA test; error bars indicate SEM.

When expressed on the surface of non-adhesive *Drosophila* S2 cells, vertebrate PrPs establish homophilic *trans*-interactions, thereby triggering the formation of weak cell contacts and subsequently accumulating at these sites [34]. Here we used this experimental paradigm to analyze how PrP protein domains and posttranslational modifications contribute to its role in cell contact formation. For this, we generated various mouse and zebrafish PrP constructs carrying deletions or point mutations, and fused them to EGFP to facilitate visualization. The mutations individually target the repetitive, hydrophobic and globular regions as well as the GPI-anchor and N-glycosylations (Fig. 1). EGFP tags were inserted immediately upstream of the repetitive domain to avoid disruption of the ER-targeting peptide and the functionally essential polybasic stretch (Fig. 1). To quantitatively assess the activity of our constructs, we determined the proportion of PrP-dependent cell-cell contacts showing PrP accumulation (Fig. 2A). In line with our previous observations, full-length, wildtype (WT) mouse PrP strongly accumulated in ∼85% of cell contacts, whereas the control construct encoding GPI-anchored EGFP (ΔCore) did so in only 3% of fortuitously formed cell contacts. Interestingly, all mutations tested clearly impaired the accumulation of PrP at contact sites, although with marked differences (Fig. 2B). For instance, removal of the GPI-anchor completely abrogated PrP activity, whereas deletion of the globular and repetitive domains dramatically reduced it (∼8% and ∼22% accumulation at cell contacts, respectively). In contrast, lack of the hydrophobic region or removal of the N-glycosylation sites led to less pronounced effects (∼65% and ∼64% accumulation, respectively). Hence, aside from confirming the essential role of the GPI-anchor, these data indicate that the repetitive and globular domains are essential for PrP accumulation at newly formed cell-cell contacts. Because constructs lacking these domains exhibit normal cell surface expression (Fig. S1A), their poor accumulation at contact sites cannot be explained by insufficient transport to the plasma membrane. More likely, these domains stabilize PrP homophilic *trans*-interactions and the associated signaling events that control further PrP clustering at contacts sites. Finally, although not as essential, the hydrophobic stretch and N-glycosylation also contribute significantly to this activity of PrP.

As observed for mouse PrP, both WT zebrafish (PrP-1 and PrP-2) proteins were found strongly enriched at S2 cell-cell contacts (∼76% and ∼94% accumulation, respectively) (Fig. 2C and D). Notably, PrP-1 and PrP-2 mutant constructs showed the same overall tendency as their mouse counterparts: deletion of GPI-anchor, globular or repetitive domains greatly affected their accumulation at cell contacts, whereas deletion of the hydrophobic region and mutation of the N-glycosylation caused less marked effects (Fig. 2C and D). These results further demonstrate that the structural determinants governing the function of PrP at cell-contacts are remarkably conserved throughout evolution.

We previously showed that the recruitment of PrP to cell-cell contacts in S2 cells is concomitant with the accumulation of F-actin and activated SFKs [34]. We now used pharmacology to determine whether PrP clustering at contact sites actually depends on cytoskeletal dynamics and SFK activity. To test for the former, S2 cells expressing mouse PrP were allowed to aggregate for 1 h and reach ∼41% PrP accumulation at cell contacts, after which the relevant inhibitors were applied (Fig. S1B). After 30 min, control DMSO-treated cells continued to aggregate and reached a two-fold increase in PrP clustering at contact sites (∼85% accumulation). Interestingly, the actin-polymerization blocker Cytochalasin D abolished and even reverted this effect (∼31% accumulation), whereas the microtubule polymerization inhibitor Nocodazole reduced it only slightly (∼75% accumulation) (Fig. S1B). Moreover, inhibition of SFK activation by PP2 also prevented PrP from clustering at cell-cell contact sites (∼41% accumulation). Similar results were obtained when the treatments were applied to zebrafish PrPs (Fig. S1C). Together, these data indicate that the accumulation of PrPs at newly formed cell-cell contacts is not only concomitant with but also dependent on actin polymerization and SFK activity.

### Targeting of PrPs to cell-cell Contacts in Human Epithelial MCF-7 Cells

**Figure 3:**
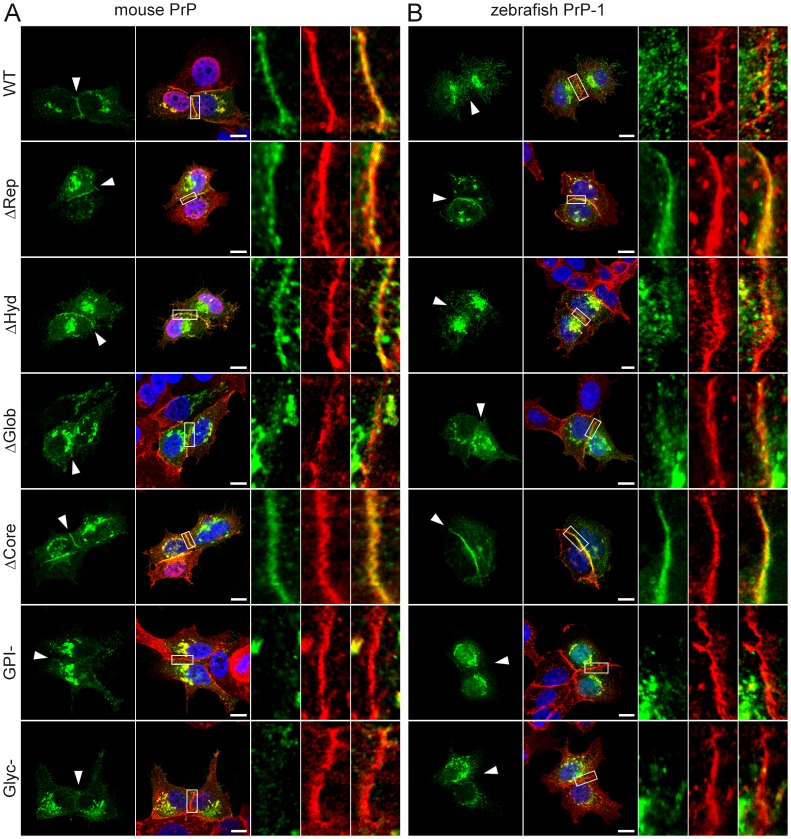
Accumulation of mouse and zebrafish PrP constructs at established MCF-7 cell cell contacts. Wild type (WT) and mutant EGFP-tagged constructs of mouse PrP (A) and zebrafish PrP-1 (B) localize differently at E-cadherin-positive cell contact sites (in red). Marked areas on the overlays are enlarged (right) to show detailed views of the contact sites. Cell nuclei are stained with DAPI (blue). Scale bars = 10 µm.

Because our experiments with S2 cells exclusively addressed the role of PrP domains in the formation of new cell contacts, we also examined the subcellular distribution of our constructs in cells with established, PrP-independent cell-cell contacts. Recent reports have shown that in MDCK epithelial cells, targeting of PrP to basolateral contact sites is primarily determined by its GPI-anchor [31], [32]. We expressed our mouse and zebrafish PrP mutant constructs in human epithelial MCF-7 cells, which form E-cadherin-dependent cell-cell contacts [40]. In these cells, WT mouse PrP clearly accumulated along contacts sites; deletion of the repetitive domain or the hydrophobic region did not affect this localization pattern (Fig. 3A). A similar distribution was observed for the control construct (ΔCore), indicating that the GPI-anchor is the minimal requirement for targeting of PrP to contact sites (Fig. 3A). Accordingly, the GPI-anchorless construct was only seen intracellularly and not associated to the plasma membrane (Fig. 3A). On the other hand, the construct lacking the globular domain accumulated poorly on isolated punctae at cell contacts despite having a functioning GPI-anchor (as indicated by its cell surface localization in S2 cells). This suggests that in MCF-7 epithelial cells, the basic targeting signal provided by the GPI-anchor is modulated by the globular domain. Notably, the unglycosylated mutant was absent from MCF-7 cell contacts (Fig. 3A), indicating that the corresponding sugar residues facilitate the GPI-dependent targeting of PrP.

The localization of zebrafish PrP-2 constructs in MCF-7 cells was remarkably similar to that of mouse PrP constructs (Fig. S2). Thus, the WT protein and deletion mutants lacking the repetitive or hydrophobic regions accumulated strongly at cell-cell contacts, whereas the ΔGlob did so only poorly, and the GPI^−^ and Glyc^−^ mutants did not at all. Interestingly, the PrP-2 control construct (ΔCore) also accumulated at cell-cell contacts like its mouse homologue (Fig. S2). In contrast, zebrafish PrP-1 constructs produced localization patterns strikingly different to those of their mouse and PrP-2 counterparts. For instance, the WT PrP-1 construct was expressed in distinct small clusters along cell-cell contacts (Fig. 3B), reminiscent of the discrete patches formed by the same construct in zebrafish embryonic cells but not in N2a or S2 cells [34]. Surprisingly, the formation of such discrete clusters was completely abrogated in the mutant lacking the repetitive domain (Fig. 3B). The accumulation of this construct along the entire cell contact indicates that the repetitive domain induces the discontinuous localization of PrP-1 in epithelial MCF-7 cells. Deletion of the hydrophobic region did not affect the local clustering of PrP-1 at cell contacts (Fig. 3B), whereas mutation of the globular, GPI or N-glycosylation sequences strongly reduced it (Fig. 3B). As reported above for mouse PrP and zebrafish PrP-2, the PrP-1 control construct also concentrated continuously along cell-cell contacts (Fig. 3B), confirming that PrP targeting to these sites is an evolutionarily conserved property encoded in the GPI-anchor.

Taken together, our results show that in an epithelial cell model, the GPI-anchor, the globular domain, and N-glycosylation positively contribute to the recruitment of mouse and zebrafish PrPs to established E-cadherin-mediated cell contacts. In addition, the repetitive domain of PrP-1 appears to induce its unique discontinuous accumulation along contact sites. These data support the notion that the localization of zebrafish PrP-2 resembles that of mammalian PrPs to a greater extent than that of its paralogue PrP-1 [34].

### Cell-surface Localization of PrPs in Early Zebrafish Embryos

**Figure 4:**
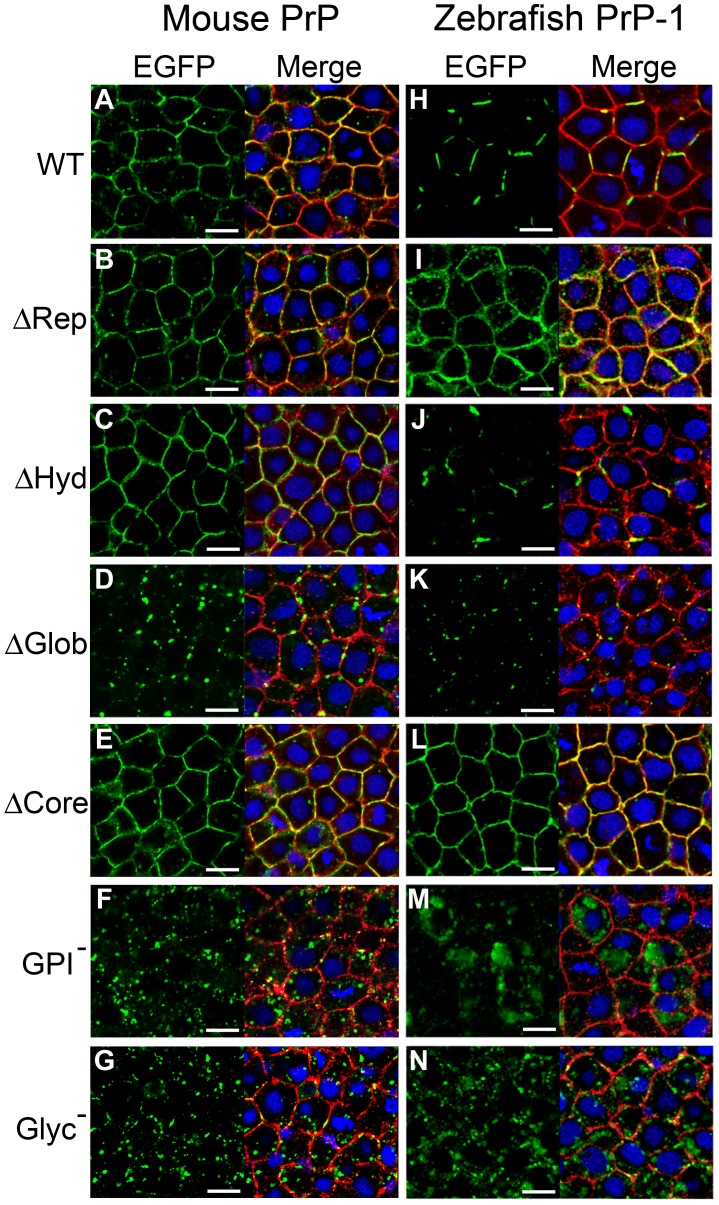
Localization of mouse PrP and zebrafish PrP-1 constructs in early zebrafish embryos. Expression of EGFP-tagged constructs (green) in the deep cells of 6 hpf zebrafish gastrulae. Plasma membranes were double-counterstained using antibodies against pY416-Src and β-catenin (merged in red). Cell nuclei were stained with DAPI (blue). Scale bars = 10 µm.

Using mRNA microinjection, we previously expressed EGFP-tagged versions of mouse and zebrafish PrPs in the deep cell layers of early fish embryos [34]. We now took advantage of this technique to examine the subcellular localization of our PrP constructs *in vivo*. In line with our initial observations, confocal analysis of 6 hpf gastrulae showed that zebrafish PrP-1 and PrP-2 are differentially expressed along the plasma membrane of deep cells: PrP-2 is distributed in a continuous pattern indistinguishable from that of mouse PrP, whereas PrP-1 accumulates locally within discontinuous patches (Fig. 4A and H; Fig. S2) [34].

Zebrafish embryonic deep cells do not form polarized epithelia but their tissue cohesion is, like that of MCF-7 cells, maintained by E-cadherin homophilic interactions [41]. Interestingly, when expressed in zebrafish embryos, mouse PrP mutants behaved similarly to what we observed in MCF-7 cells. For instance, deletion of the repetitive domain or the hydrophobic region did not affect its continuous distribution along the plasma membrane (Fig. 4B and C). In contrast, deletion of the globular domain caused the construct to accumulate in punctate structures at the plasma membrane (Fig. 4D). At first glance, this result may suggest that the globular domain is essential for localization of PrP along the entire plasma membrane. However, we excluded this possibility because the control deletion mutant, which lacks the entire protein core (including the globular domain), localized like the WT protein (Fig. 4E). Thus, the leader peptide and GPI-anchor are sufficient to ensure continuous surface expression of PrP. Most likely, either the repetitive domain or the hydrophobic regions are responsible for the punctate distribution of the ΔGlob mutant, and the globular domain counteracts this effect. On the other hand, the GPI-anchorless and unglycosylated constructs were expressed poorly at the plasma membrane, accumulating instead intracellularly (Fig. 4F and G). The localization patterns of these mutants confirm that the GPI-anchor is necessary for tethering PrP to the plasma membrane *in vivo*, and further indicate that N-glycosylation plays an important role during trafficking and/or sorting of PrP to cell contact sites. As seen in MCF-7 cells, PrP-2 and mouse PrP mutant constructs localized similarly in zebrafish embryos. Particularly, removal of the globular domain, GPI-anchor and glycosyl residues caused the same distinct effects on PrP-2 localization (Fig. S2).

Further analyses were conducted with zebrafish PrP-1 in order to study its distinctive patchy distribution. These experiments revealed interesting differences and similarities with mouse PrP and zebrafish PrP-2. For instance, deletion of the repetitive domain had the same effect on PrP-1 localization seen in MCF-7 cells, as the corresponding construct was expressed continuously along the plasma membrane (Fig. 4I). Thus, the extensive repetitive domain of PrP-1 is required for its local accumulation in patches at the plasma membrane. Deletion of the PrP-1 hydrophobic region or the globular domain had the same effect as seen with mouse PrP and zebrafish PrP-2 constructs: lack of the hydrophobic region did not alter PrP-1′s patchy localization (Fig. 4J), whereas absence of the globular domain induced the punctate accumulation of PrP-1 at the plasma membrane (Fig. 4K). Moreover, the latter effect was observed only when the repetitive domain was present (ΔGlob mutant) but not when the entire protein core was deleted (ΔCore mutant, Fig. 4L). These results further suggest that the repetitive and globular domains exert opposing effects on the patterned distribution of PrP within contact sites. Finally, as seen with mouse PrP and zebrafish PrP-2 constructs, lack of GPI-anchoring or N-glycosylation also caused PrP-1 to localize poorly at the plasma membrane and remain intracellular (Fig. 4M and N). In particular, extensive intracellular accumulation was observed for the GPI-anchorless mutant (Fig. 4M).

### Roles of PrP During Gastrulation

**Figure 5:**
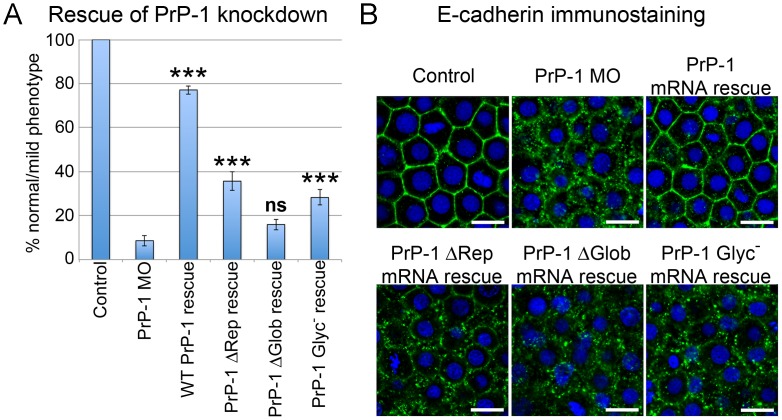
Rescue of PrP-1 knockdown embryos by mutant PrP-1 constructs. PrP-1 morphant embryos were microinjected with mRNAs encoding selected EGFP-tagged PrP-1 constructs, and their rescue activity was evaluated morphologically and molecularly. A) Quantitative differences in rescue activity between untreated (control) or morphant embryos (PrP-1 MO), and embryos expressing WT, ΔRep, ΔGlob and Glyc^−^ PrP-1 constructs. Data are given as the proportion of embryos showing normal-to-mild gastrulation phenotypes at 6 hpf. Three independent experiments were analyzed (average n = 30 embryos). Triple asterisks [***] indicate statistically significant rescues at *p*<0.001; one-way ANOVA test; error bars represent SEM. B) Confocal images of deep cells from embryos immunostained against E-cadherin. Rescue is indicated by the recovery of E-cadherin cell-surface localization. Scale bar = 10 µm.

To determine whether the PrP mutant constructs showing abnormal localization patterns were functionally impaired, we tested their ability to rescue our previously described PrP-1 knockdown phenotype [34]. To simplify our analysis, only the following PrP-1 constructs were tested: WT (patchy localization, positive control), ΔRep (continuous localization), ΔGlob (punctate distribution) and Glyc^−^ (intracellular accumulation). The corresponding mRNAs were co-injected at the one-cell stage with PrP-1 morpholino, and the embryos were scored at 6 hpf (50% epiboly). Differences between constructs were quantified as the proportion of embryos able to overcome the PrP-1 knockdown gastrulation arrest and carry out epiboly (Fig. 5A). These experiments showed that, unlike WT PrP-1 (77.15% rescued embryos), the mutant constructs had a significantly reduced ability to revert the knockdown phenotype. This reduction was more pronounced for the ΔGlob construct (no significant rescue) than for the ΔRep construct (35.67% rescued embryos), in apparent correspondence with the strongly abnormal localization pattern of the former. Interestingly, while deletions of the repetitive or globular domains had opposite effects on PrP-1 localization (continuous vs. punctate distributions at the plasma membrane, respectively), both of them negatively affected PrP-1 function in epiboly. Similarly, mutation of the N-glycosylation led to a considerable decrease in rescuing activity (28.24% rescued embryos), possibly due to its poor cell surface expression.

As reported in our earlier work, PrP-1 knockdown embryos exhibit defects in cell-cell adhesion due to the progressive loss of E-cadherin from cell contacts [34]. Therefore, we tested the same constructs for their ability to specifically rescue this defect. Whole-mount immunostaining showed that WT PrP-1 mRNAs could restore the normal surface localization of E-cadherin, whereas the ΔRep, ΔGlob and Glyc^−^ mutants largely fail to do so (Fig. 5B). Hence, the ability of PrP-1 to stabilize E-cadherin at cell contacts correlates with its discontinuous, patchy localization at the plasma membrane, which appears to be dictated by the interplay between the repetitive and globular domains (see above). Similarly, the predominantly intracellular localization pattern of the N-glycosylation mutant correlates with its failure to restore E-cadherin at cell-cell contacts.

**Figure 6:**
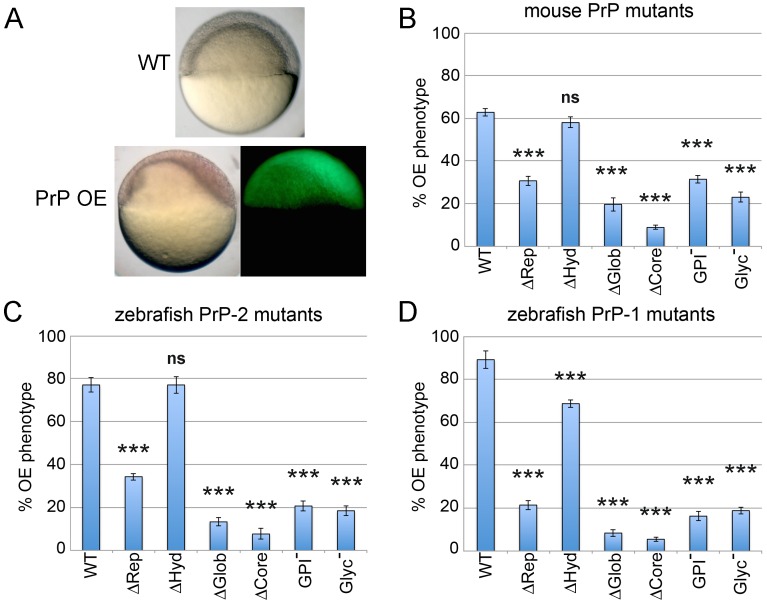
Overexpression (OE) of mouse and zebrafish PrP constructs in early zebrafish embryos. Embryos were microinjected with mRNAs encoding all mouse and zebrafish EGFP-tagged PrP constructs. A) OE of mouse or zebrafish WT PrPs in early embryos produces a gain-of-function phenotype characterized by asymmetric gastrulation at 6 hpf. B–D) The activities of mouse and zebrafish constructs were evaluated morphologically by quantifying the proportion of 6 hpf embryos exhibiting the OE phenotype (asymmetric gastrulation). Three independent experiments were analyzed (average n = 30 embryos). Triple asterisks [***] indicate statistically significant reduction in activity at *p*<0.001; one-way ANOVA test; error bars represent SEM.

To further assess the physiological relevance of these changes *in vivo*, we took advantage of a PrP gain-of-function phenotype previously described in zebrafish gastrulae [34]. In that study, overexpression of WT zebrafish or mouse PrPs induced a clear morphological phenotype characterized by asymmetric epiboly in the majority of embryos at 6 hpf (Fig. 6A). Therefore, we asked how the mutations introduced in our constructs would affect this activity of PrPs. As functional readout, we quantified the number of embryos showing overexpression (OE) phenotypes upon expression of each construct (Fig. 6B–D). Interestingly, analogous deletions in mouse or zebrafish PrPs produced similar changes in their activity (Fig. 6B–D). For instance, compared to WT constructs, all three mutants lacking the hydrophobic stretch retained significant activity (∼92%, ∼100% and ∼77% for mouse PrP, zebrafish PrP-2 and zebrafish PrP-1, respectively). In contrast, deletion of the globular or repetitive domains significantly reduced the ability of these mutants to cause the embryonic OE phenotype. Moreover, the activity of the globular domain mutants was consistently lower than that of repetitive domain ones (∼31% vs ∼49% for mouse PrP, ∼17% vs ∼45% for zebrafish PrP-2 and ∼9% vs ∼24% for zebrafish PrP-1). On the other hand, mutants lacking the entire protein core were only minimally able to elicit OE phenotypes (∼14%, ∼10% and ∼6% activity for mouse PrP, zebrafish PrP-2 and zebrafish PrP-1, respectively). Notably, the levels of activity of unglycosylated PrPs were comparable to those of the ΔGlob mutants (∼37%, ∼24% and ∼21% for mouse PrP, zebrafish PrP-2 and zebrafish PrP-1 mutants, respectively). Since PrP N-glycosylation sites are located within the globular domain, this result further indicates that sugar residues are key functional elements of this domain. Finally, GPI-anchorless mutants showed residual levels of activity larger than those of ΔCore mutants (∼50%, ∼27% and ∼18% for mouse PrP, zebrafish PrP-2 and zebrafish PrP-1, respectively), suggesting that they carry out functional interactions (possibly with endogenous PrP-1) despite not being tethered to the plasma membrane.

## Discussion

Determining the physiological significance of PrP requires thorough understanding of how its structural elements contribute to its activity and distribution in different cells. Here we examined this subject using two functional readouts developed in a previous study [34]. First, when exogenously expressed in insect and mammalian cells, PrP accumulates at cell-cell contacts, promoting cell adhesion and intracellular signaling. Second, PrP-1 knockdown in early zebrafish embryos reduces cell surface expression of E-cadherin, resulting in lethal gastrulation arrest. Notably, mouse and zebrafish PrPs share the ability to establish homophilic *trans*-interactions, trigger SFK signaling, rescue the PrP-1 knockdown phenotype, and cause the same developmental gain-of-function phenotype in zebrafish [34]. This evolutionary conservation and the genetic tractability of the zebrafish embryo make it an ideal model to carry out functional mutagenesis of PrP *in vivo*.

When expressed in *Drosophila* S2 cells, all our mouse and zebrafish constructs -with exception of the GPI-anchorless mutants- displayed cell surface localization. Interestingly, even mutation of both Asn N-glycosylation residues to Gln (GlnAsn) left delivery of PrP to the plasma membrane of these cells unaffected. This is in line with a previous report showing that WT hamster PrP is correctly targeted to the surface of S2 cells despite being incompletely glycosylated [42]. In contrast, the same glycosylation mutants did not reach the plasma membrane when expressed in MCF-7 cells or zebrafish embryos. Similar results have been reported using the same and other glycosylation mutants in various mammalian cell lines [29], [32], [43]–[45]. Importantly, treatment of CHO and human neuroblastoma cells with tunicamycin efficiently blocks PrP glycosylation without affecting its trafficking [29], [46]. Together with more detailed mutational analyses [47], these studies strongly suggest that most mutations of the consensus sequence Asn-X-Thr (and not the lack of glycosylation *per se*) result in deficient PrP trafficking in vertebrate cells. The reason for this is unclear, as is the normal trafficking of these mutants in *Drosophila* cells. Because N-linked glycans are important to ensure proper folding, stability and quality control of glycoproteins in the ER [48], these observations may be explained by differences in biosynthetic processing and folding of proteins between vertebrate and invertebrate cells.

In our *Drosophila* cellular model, membrane-bound PrPs establish homophilic *trans*-interactions leading to cell contact formation, SFK activation and actin cytoskeletal rearrangements [34]. Here we used pharmacological inhibitors to demonstrate that the subsequent accumulation of additional PrP molecules at contact sites requires SFK activity as well as remodeling of the actin cytoskeleton. These data are indicative of a self-regulatory mechanism in which PrP homophilic *trans*-interactions trigger intracellular signals that enhance the recruitment of more PrP to cell-contact sites. Accordingly, deletion of either two of the largest PrP domains exposed on the cell surface -globular and repetitive- significantly compromised the conserved ability of mouse and fish PrPs to *trans*-interact and cluster at cell contacts. These results are in agreement with the previous identification of these two regions as PrP/PrP interaction domains using a yeast two-hybrid system [49]. Such a strong effect was not observed upon removal of the hydrophobic domain, suggesting that this small region does not significantly contribute to the ability of PrP to form contacts sites and/or trigger intracellular signals.

In certain epithelial cell types, surface expression of PrP is particularly evident at contact sites and basolateral membranes [50], [51]. In mouse neuroblastoma N2a, HeLa [34] and MCF-7 cells (this study), fish and mammalian PrPs similarly accumulate at cell-cell contacts. The targeted sorting of PrP is determined by a molecular signal encoded within the GPI-anchor and modulated by N-linked glycans [31], [32], although an earlier report ascribed this role to the hydrophobic domain [52]. In our hands, the hydrophobic domain did not appear to play a role in targeting of PrP to MCF-7 cell contacts, whereas the GPI-anchor and N-glycosylation were essential for it. Interestingly, while the globular domain also proved necessary for targeted sorting of PrP to contact sites, the repetitive domain appeared to be dispensable. This contrasts with the situation in S2 cells, where both domains were required for PrP accumulation at contact sites. This difference is likely due to the fact that the formation of S2 cell contacts requires the establishment of PrP *trans*-interactions, whereas MCF-7 cell contacts are independently maintained by E-cadherin homophilic interactions [53]. Therefore, PrP accumulation at MCF-7 contact sites does not depend on its ability to *trans*-interact (via the globular and repetitive domains), but on its targeted sorting, which is controlled by the GPI-anchor signal and N-glycans at the globular domain (but not the repetitive domain). Our finding that the GPI-anchor signal is sufficient to drive the accumulation of an EGFP construct to MCF-7 cell contacts is in line with similar experiments carried out in MDCK cells [31], [32]. However, we did not observe this phenomenon in N2a or HeLa cells [34], which form E-cadherin-independent contacts [54], [55]. The reason for this discrepancy is unclear but it may suggest that the targeted sorting of PrP to cell contacts is positively modulated by cell-type-specific components in addition to the GPI-anchor signal.

The localization patterns of our constructs in MCF-7 cells strongly resemble our previous observations in living zebrafish embryonic cells [34]. In both systems, WT mouse PrP and zebrafish PrP-2 exhibit a continuous distribution along cell-cell contacts, whereas WT PrP-1 accumulates only at discrete subregions of the contact site. These local clusters of PrP-1 vary in size, ranging from dot-like structures in MCF-7 cells to elongated patches in ZF embryonic cells. Here we show that the unique, discontinuous distribution of PrP-1 is caused by its repetitive domain, as deleting only this region restores continuous localization of the constructs along cell-cell contacts. Notably, removal of the globular domain in all three PrPs (PrP-1, PrP-2 and mouse PrP) produces a similarly punctate, discontinuous distribution, both in MCF-7 and in zebrafish embryonic cells. Two key conclusions can be derived from these observations. First, that the repetitive domain has the conserved property of promoting the local accumulation of PrP at specific subregions within a contact site. Second, that the globular domain counteracts this effect, possibly by stabilizing PrP interactions along larger regions of the contact site. It is not clear from our experiments whether the repetitive domain induces the discontinuous distribution of PrP by promoting its local clustering at discrete locations within the contact site, or by facilitating its exclusion from other, complementary subregions of the cell contact. The former scenario is in line with earlier studies ascribing self-aggregation properties to the repetitive region [56], [57], whereas the latter one may be related to its reported role in copper-dependent PrP endocytosis [25]. Interestingly, the presence of copper-binding histidines is not conserved in the repetitive regions of zebrafish PrPs [13], [14], [58], suggesting that the copper-binding activity of mammalian PrP is evolutionarily acquired and not related to a conserved PrP function. In fact, the differences that we see in localization/activity of our N-terminal mutants are conserved in fish and mammalian constructs, irrespective of the presence or absence of histidines. The fact that WT PrP-1 normally localizes in punctae/patches would suggest that its repetitive region has a stronger clustering activity than that of mouse PrP or PrP-2. In fact, PrP-1 contains larger and more complex repeats than mouse PrP or PrP-2, owing to multiple expansion cycles of this domain during evolution [13], [58]. Thus, its globular domain would not be sufficient to ensure a continuous localization pattern. These assumptions can be experimentally tested using chimeric constructs.

Using zebrafish embryonic phenotypes as functional readouts [34], we found a clear correspondence between PrP protein domains, localization at cell contacts, and *in vivo* activity. In our mRNA rescue experiments, three PrP-1 mutations causing distinct abnormal localization patterns −ΔRep (continuous, cell surface), ΔGlob (punctate, cell surface) and Glyc^−^ mutants (largely intracellular)- failed to revert the gastrulation arrest phenotype caused by PrP-1 knockdown. Moreover, we could show that their functional impairment is associated with their inability to restore E-cadherin to the plasma membrane of deep cells. The poor rescuing activity of PrP-1 mutants like ΔGlob and Glyc^−^ could be in part explained by their low cell surface expression. However, the ΔRep mutant also exhibits a strongly reduced activity despite being expressed over the entire plasma membrane. Hence, these results most likely reflect the relative contributions of both the repetitive and globular domains to the stability of PrP-1 homophilic *trans*-interactions and their downstream signaling. It remains to be established whether the local clusters of PrP-1 at cell contacts define signaling subregions of the plasma membrane, or whether they result from the specific recruitment of PrP-1 to preformed specialized sites. Interestingly, a similar localization pattern has been described for zebrafish Frizzled 7 (Fz7), and shown to modulate the persistence of cell contacts in the gastrula [59]. In that study, non-canonical Wnt11 was found to induce the local accumulation of Fz7 at “adhesive subdomains” within contact sites, defined by the presence of the atypical cadherin Flamingo (Fmi). Additionally, Wnt11 can modulate cell adhesion in the zebrafish gastrula by inducing the endocytosis and recycling of E-cadherin [60]. Our finding that PrP-1 accumulates locally at cell contacts to regulate E-cadherin turnover and embryonic cell adhesion suggests interesting parallels with the Wnt11/Fz7/Fmi model. A related phenomenon was reported in HeLa cells and *Xenopus* animal caps, where canonical Wnt induces the local aggregation of LRP6-signalosomes at cell contacts to stabilize β-catenin [61]. Whether these similarities are purely mechanistic or whether the PrP-1 and Wnt signaling pathways are indeed functionally interconnected remain exciting questions.

By overexpressing mutant constructs in zebrafish embryos, we quantitatively confirmed that the functional contributions of PrP domains are conserved between mouse and zebrafish. In our experiments, deletion of the central hydrophobic region did not alter mouse or fish PrP localization and affected only minimally the ability of these constructs to cause a developmental gain-of-function phenotype. This stands in contrast with earlier studies in transgenic mice where related deletions suggested the existence of a functional domain involved in preventing neurotoxic signals [26], [27]. However, it should be noted that two of those deletions -PrPΔ_CD_ and PrPΔ_CR_, mouse aa pos. 94–134 and 105–125, respectively- are longer than ours and extend totally or partially into the N-terminally-located charged cluster 1 (CC_1_), which is not deleted in our constructs (mouse aa pos. 112–126). Based on comparisons of all vertebrate PrP sequences available [13], we chose positions 112–126 to examine the role of the most conserved region of the hydrophobic core. Therefore, the neuropathological signs observed in transgenic mice are likely due to deletion of residues within the CC_1_ region. In fact, a smaller deletion reported previously, and enclosed within ours -PrPΔ_114–121_ [26]- is not toxic but shows instead a reduced ability (relative to WT PrP) to rescue the toxic phenotype caused by introduction of PrPΔ_32–134_. Alternatively, the neurotoxicity of the PrPΔ_CD_ and PrPΔ_CR_ mutants may require additional factors not expressed in early fish embryos. In fact, transgenic mice carrying these deletions do not exhibit developmental problems, and neurotoxicity is not apparent until later adult stages [26], [27]. Generation of stable transgenic fish expressing these constructs in neuronal tissues should help clarify this matter.

On the other hand, mutations affecting PrP cell surface expression in MCF-7 cells or its ability to *trans*-interact in S2 cells -namely GPI^−^, Glyc^−^ and ΔGlob- strongly suppressed the zebrafish overexpression phenotype. While such a suppressive effect was also evident in the ΔRep mutants, quantification of the mRNA rescue and overexpression experiments indicate that they retain higher levels of *in vivo* activity than the ΔGlob mutants. These data suggest that the conserved PrP function recorded in our zebrafish assays is encoded in the globular domain, and that the repetitive domain enhances this activity. A similar conclusion was reached regarding the role of the repetitive domain in prion pathogenesis and replication [62]. Nevertheless, in uninfected transgenic mice, expression of N-terminally deleted PrPs triggers neurodegenerative phenotypes [63]–[65]. Other reports found that the octarepeats are dispensable when rescuing such toxic phenotypes with PrP constructs [26], [66]. Interestingly, one of these studies showed that octarepeat expansion impairs rescuing activity [66], whereas a later one concluded that the N-terminus is necessary and sufficient for PrP function [67]. Hence, although the data from transgenic mice are complex and apparently paradoxical, our own results are in line with the scenario of a functionally important repetitive domain. It is of note that the largest reduction of *in vivo* activity among ΔRep mutants was recorded for the PrP-1 mutant. Interestingly, the ΔRep deletion affects the unique localization pattern of PrP-1 but not those of PrP-2 or mouse PrP, suggesting that the patchy distribution of PrP-1 along cell contacts is key to its function in the early embryo.

Taken together, the present data confirm the essential requirement of the GPI-anchor and N-glycosylations for PrP localization and function of fish and mammalian PrPs. In addition, they uncover a conserved interplay between the repetitive and globular domains, which modulates the local accumulation of PrP at cell-cell contacts and its ability to regulate cell-cell interactions. Further biochemical and cell biological studies across model organisms are needed to relate PrP domains with specific molecular pathways *in vivo*. The suitability of the zebrafish as a neurodevelopmental model, its ease of manipulation and the possibility to analyze signaling in early embryos offer a great opportunity to decipher PrP-associated signaling networks involved in neuronal physiology.

## Supporting Information

Figure S1 **Accumulation of mouse and zebrafish PrPs at newly formed cell contacts in** ***Drosophila*** **S2 cells requires the actin cytoskeleton and SFK activity.** A) Accumulation of mouse EGFP-PrP deletion mutants at cell contacts in *Drosophila* S2 cells. Expression of mouse PrP lacking the repetitive (m PrP ΔRep) and the globular (m PrP ΔGlob) domains induce cell contact formation with a reduced accumulation of PrP at contact sites. Note that both PrP deletion mutants were normally expressed at the plasma membrane. Scale bars = 5 µm. B,C) Quantification of the effect of Cytochalasin D (Cyt D), Nocodazol and PP2 in the number of transfected S2 cell contacts showing accumulation of wild type mouse PrP (B) and zebrafish PrP-2 (C). S2 cells were allowed to form contacts for 60 min previous to a treatment with DMSO, Nocodazol or Cyt D for additional 30 min. After this time, control DMSO-treated cells continue aggregating PrP at cell contacts whereas Cyt D-treated cells failed to further accumulate PrP. A slight reduction of PrP accumulation as contacts was observed in Nocodazol-treated cells. Alternatively, PrP expressing S2 cells were treated with DMSO or PP2 for additional 120 min. Inhibition of SFKs by PP2 blocked further accumulation of PrP at cell contact sites (n = 6, *p<0.05, ***p<0.001, one-way ANOVA test; error bars indicate SEM).
(TIF)

Figure S2 **Localization of zebrafish PrP-2 at cell-cell contacts in epithelial MCF-7 cells and 6 hpf zebrafish deep cells.** Expression of zebrafish PrP-2 EGFP fusion wild type (WT) and mutant (indicated in the figures) constructs localized differentially at cell contact sites (white arrowheads). Scale bars = 10 µm.
(TIF)
