# Supplementary figures and images for: Conserved Roles of the Prion Protein Domains on Subcellular Localization and Cell-Cell Adhesion
Source: PLoS One. 2013 Jul 31;8(7):e70327. doi: 10.1371/journal.pone.0070327 (PMC3729945; doi:10.1371/journal.pone.0070327)

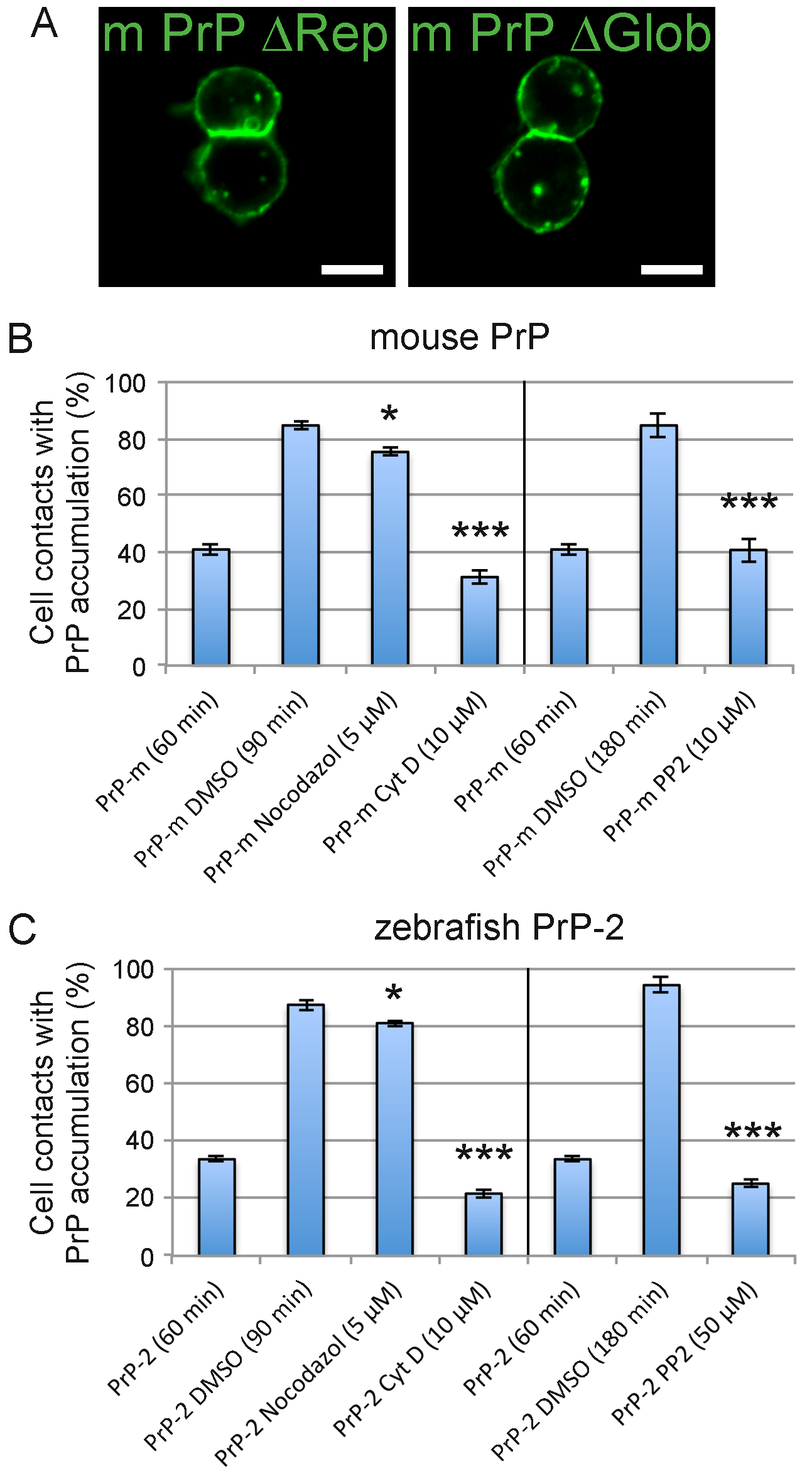

Supplement: Figure S1 — Accumulation of mouse and zebrafish PrPs at newly formed cell contacts in Drosophila S2 cells requires the actin cytoskeleton and SFK activity. A) Accumulation of mouse EGFP-PrP deletion mutants at cell contacts in Drosophila S2 cells. Expression of mouse PrP lacking the repetitive (m PrP ΔRep) and the globular (m PrP ΔGlob) domains induce cell contact formation with a reduced accumulation of PrP at contact sites. Note that both PrP deletion mutants were normally expressed at the plasma membrane. Scale bars = 5 µm. B,C) Quantification of the effect of Cytochalasin D (Cyt D), Nocodazol and PP2 in the number of transfected S2 cell contacts showing accumulation of wild type mouse PrP (B) and zebrafish PrP-2 (C). S2 cells were allowed to form contacts for 60 min previous to a treatment with DMSO, Nocodazol or Cyt D for additional 30 min. After this time, control DMSO-treated cells continue aggregating PrP at cell contacts whereas Cyt D-treated cells failed to further accumulate PrP. A slight reduction of PrP accumulation as contacts was observed in Nocodazol-treated cells. Alternatively, PrP expressing S2 cells were treated with DMSO or PP2 for additional 120 min. Inhibition of SFKs by PP2 blocked further accumulation of PrP at cell contact sites (n = 6, *p<0.05, ***p<0.001, one-way ANOVA test; error bars indicate SEM). (TIF) [file pone.0070327.s001.tif]

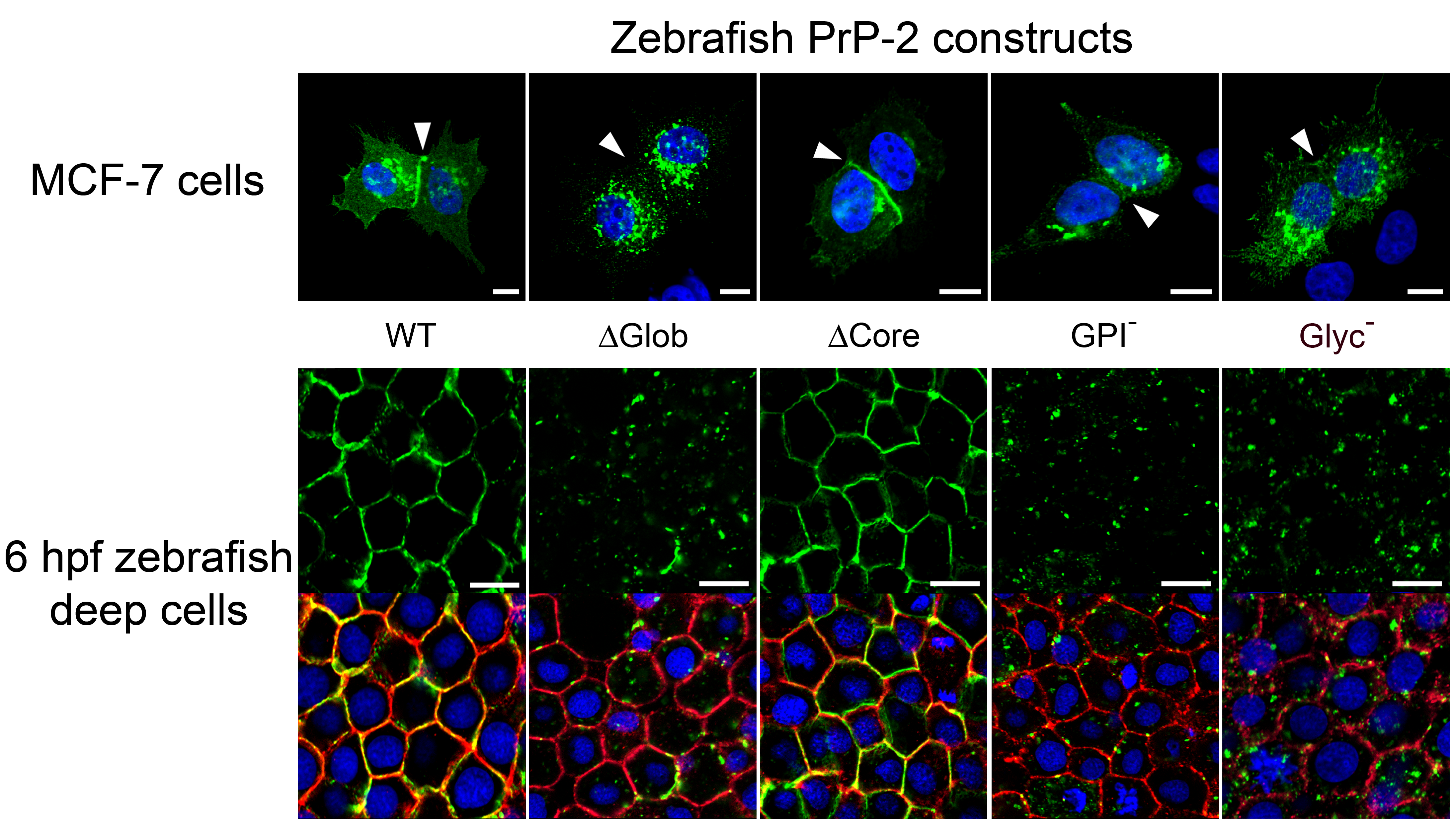

Supplement: Figure S2 — Localization of zebrafish PrP-2 at cell-cell contacts in epithelial MCF-7 cells and 6 hpf zebrafish deep cells. Expression of zebrafish PrP-2 EGFP fusion wild type (WT) and mutant (indicated in the figures) constructs localized differentially at cell contact sites (white arrowheads). Scale bars = 10 µm. (TIF) [file pone.0070327.s002.tif]
